# Caspase-11 Non-canonical Inflammasomes in the Lung

**DOI:** 10.3389/fimmu.2020.01895

**Published:** 2020-08-21

**Authors:** Changhoon Oh, Ambika Verma, Youssef Aachoui

**Affiliations:** Department of Microbiology and Immunology, Center for Microbial Pathogenesis and Host Responses, University of Arkansas for Medical Sciences, Little Rock, AR, United States

**Keywords:** caspase-11, gasdermin D, inflammasomes, pore intracellular traps (PIT), *Burkholderia thailandensis* lung defense, Gbps, LPS

## Abstract

The airway epithelium and underlying innate immune cells comprise the first line of host defense in the lung. They recognize pathogen-associated molecular patterns (PAMPs) using membrane-bound receptors, as well as cytosolic receptors such as inflammasomes. Inflammasomes activate inflammatory caspases, which in turn process and release the inflammatory cytokines IL-1β and IL-18. Additionally, inflammasomes trigger a form of lytic cell death termed pyroptosis. One of the most important inflammasomes at the host-pathogen interface is the non-canonical caspase-11 inflammasome that responds to LPS in the cytosol. Caspase-11 is important in defense against Gram-negative pathogens, and can drive inflammatory diseases such as LPS-induced sepsis. However, pathogens can employ evasive strategies to minimize or evade host caspase-11 detection. In this review, we present a comprehensive overview of the function of the non-canonical caspase-11 inflammasome in sensing of cytosolic LPS, and its mechanism of action with particular emphasis in the role of caspase-11 in the lung. We also explore some of the strategies pathogens use to evade caspase-11.

## Introduction

Bacterial infections of the respiratory tract remain a leading cause of global morbidity and mortality (1). As the first line of defense against infection, the airway epithelium and innate immune cells play a critical role in limiting infection. As such, they are especially sensitive to pathogen associated molecular patterns (PAMPs), expressing an array of membrane-bound and cytosolic receptors that recognize specific structural motifs shared by classes of microbes (2). For example, Toll-like receptor 5 (TLR5) and NOD-like receptor 4 (NLRC4) detect and respond to bacterial flagellin. TLR5 detects extracellular flagellin (3), while NLRC4 detects its cytosolic presence (4, 5).

Many pathogens infect cells and exploit intracellular niches to facilitate their replication and spread (6). In turn, host cells have evolved mechanisms to detect and counteract these efforts. For example, intracellular sensors like inflammasomes are expressed in innate immune cells, lung epithelium, and endothelium to enable detection of pathogens and subsequent protective responses (6–8). Inflammasomes lead to the activation of inflammatory caspases, which in turn process and release the inflammatory cytokines IL-1β and IL-18. Additionally, inflammasomes trigger a form of lytic cell death termed pyroptosis that plays important role in clearance of offending pathogens (6).

Virulent intracellular bacteria usually evade intracellular sensors or suppress inflammasome activity by suppressing or modifying the structural features of their PAMPs (9). These strategies diminish the ability of inflammasomes to respond to these pathogens. For instance, *Salmonella enterica* serovar typhimurium utilizes two pathogenicity islands, SPI-1 and SPI-2, which encode distinct type three secretion systems (T3SSs). The T3SS apparatus encoded by SPI-1 functions primarily during epithelial cell invasion, is co-expressed with flagellin, and is readily detected by the inflammasome NLRC4. In contrast, the SPI-2 T3SS is expressed in the vacuolar compartment of both epithelial cells and macrophages, and is not detected by inflammasomes. *S. typhimurium* suppresses transcription of flagellin during SPI-2 inducing conditions, thereby avoiding detection by NLRC4 (10, 11). Here, we present a comprehensive overview of the function of the caspase-11 inflammasome in sensing of cytosolic LPS, and its mechanism of action with particular emphasis in the lung. We also explore some of the strategies pathogens use to evade caspase-11.

## Inflammasomes Activate Inflammatory Caspase-1 and -11

Caspases are a family of aspartate-specific, cysteine proteases that are activated by homodimerization or cleavage to mediate several cellular events including cell death (12). Cell death is an essential process that maintains tissue homeostasis, and recently, it has emerged as an important defense mechanism against infection (13). Caspases are divided into inflammatory (in mouse these include caspase-1,-11, and -12) or apoptotic caspases (caspase-2,-3,-6,-7,-8, and -9). Apoptotic caspases are further subdivided into initiators (caspase-2,-8, and -9) or executioners (caspase-3,-6, and -7) (12). A discussion of the activation and role of apoptotic caspases during infection is important; however, it is beyond the scope of this review, and we refer the reader to previously published reviews (12).

Inflammatory caspases are able to distinguish virulent from avirulent bacteria, and to alert the immune system to a pathogenic infection. Like apoptotic initiator caspases, inflammatory caspases contain a death fold prodomain termed the caspase activation and recruitment domain (CARD) (12). The CARD domain is essential to the recruitment and activation of inflammatory caspases-1 and-11 within the inflammasomes. Inflammasomes are large, multiprotein complexes typically comprising a NOD-like receptor (NLR) or AIM2-like receptor (ALR) such as NLRP3, NLRC4, or AIM2, sometimes an adaptor protein called ASC, and an executioner caspase, which is typically caspase-1 (6). Several inflammasomes that activate caspase-1 in response to contamination of the cytosol or perturbation of cellular physiology have been characterized, and each one is named after its NLR or ALR protein scaffold. The NLRP3 inflammasome is activated in response to the widest array of stimuli, including particulates, crystals, ATP, K^+^ efflux, lysosomal disruption, and oxidized mitochondrial DNA. However, the precise signal that NLRP3 detects remains controversial (14). In contrast to the diverse stimuli that activate NLRP3, other inflammasomes respond to a limited set of stimuli. For instance, The AIM2 detects cytosolic dsDNA (15–17), which may be encountered in the cytosol during pathogenic infection. The NLRC4 inflammasome responds to flagellin and the activity of type III secretion system (T3SS) and type IV secretion system (T4SS) (10, 18, 19). Unlike other inflammasomes, activation of NLRC4 involves members of the NAIP subfamily of NLRs, forming mixed NLRC4-NAIP oligomeric inflammasome platforms. The murine NAIP locus is highly polymorphic. C57BL/6 mice encode four NAIPs; NAIP1 binds the T3SS needle, NAIP2 binds the T3SS rod, and NAIP5 and NAIP6 bind flagellins. By contrast, humans have a single NAIP that detects the T3SS needle (18–21). Upon activation, NLRs forming inflammasomes recruit the ASC adaptor, composed of a CARD and a PYD, via homotypic interactions. Additional ASC molecules are incorporated via CARD-CARD and PYD-PYD interactions, until all ASC are collected into a single focus called a “speck.” Attraction of caspase-1 into the ASC speck via CARD-CARD interaction results in caspase-1 dimerization and proximity-induced autoproteolytic processing into p10 and p20 subunits (6). Active caspase-1 processes pro-IL-1β and pro-IL-18 to their mature forms; it additionally triggers a lytic form of programmed cell death called pyroptosis by cleaving and activating the pore-forming protein gasdermin D (GSDMD; discussed in more detail below) (6, 22). Activation of inflammasomes can also promote the release of IL-1α. IL-1α is considered an endogenous danger signal that is widely and constitutively expressed, active in its uncleaved form, and passively released after pyrpotosis (23).

Caspase-1 was long thought to be the only inflammatory caspase activated within an inflammasome and the sole mediator of pyroptosis because caspase-1-deficient bone marrow macrophages (BMMs) were resistant to inflammasome activation-induced cell death. However, the Dixit lab showed that the process of backcrossing the caspase-1 knockout allele into the C57BL/6 background carried a passenger mutation in caspase-11 from the 129 background, effectively making *Casp1-Casp11*^*DKO*^ animals (24). Examination of the single *Casp11*^−/−^ mice revealed that, caspase-11 drives pyroptosis upon *in vitro* activation like the closely related caspase-1, but it does not directly mediate IL-1β and IL-18 secretion. Instead, caspase-11 can activate an indirect NLRP3-ASC-caspase-1 pathway leading to IL-1β/IL-18 processing and release (24). The caspase-11 pathway was thus termed the “caspase-11 non-canonical inflammasome” to contrast it with the caspase-1-activating canonical inflammasomes (NLRP3, NLRC4, AIM2, etc.). These revelations ignited interest into the study of caspase-11.

## Caspase-11 Directly Detects the Cytosolic Presence of LPS

**Figure 1:**
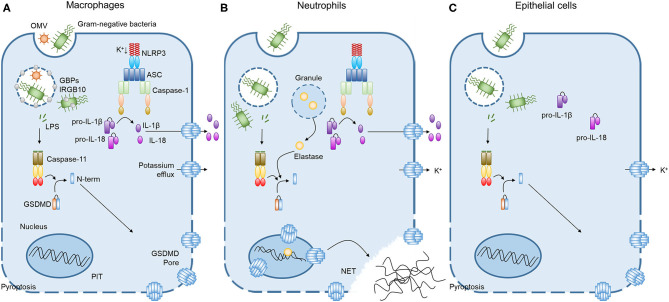
Caspase-11 non-canonical inflammasome activation by intracellular LPS in different cells. **(A)** Cooperation of GBPchr3 with IRGB10 expose LPS from intracellular bacteria or OMVs in macrophages. Cytoplasmic LPS directly bind to the CARD motif of caspase-11 with their lipid A moiety, leading to oligomerization of caspase-11 to activate the non-canonical inflammasome. Active caspase-11 cleaves GSDMD to release its N-terminal domain, which is subsequently inserted into plasma membrane to form membrane pores. Potassium efflux through GSDMD pores additionally triggers NLRP3 inflammasome, inducing the processing and release of IL-1β and IL-18. Pyroptosis pores in the plasma membrane are large enough to permit soluble proteins to defuse to the extracellular milieu, but small enough to trap organelles and intracellular bacteria. This process is termed the pore-induced intracellular trap (PIT). **(B)** In neutrophils, both caspase-11 and elastase can cleave GSDMD to trigger neutrophil extracellular traps (NETs). Cleaved GSDMD targets the nucleus in addition to the plasma membrane and drives nuclear permeabilization, chromatin relaxation and plasma membrane rupture in elastase or caspase-11 dependent manner. These processes induce neutrophil to extrude NETs which restrict bacterial replication, dissemination, and enhance bacteria killing. **(C)** In contrast to the broadly expressed caspase-11 and GSDMD, caspase-1 inflammasome components are not detectable in lung epithelial cells. Therefore, the caspase-11 non-canonical inflammasome activation in lung epithelial cells only triggers pyroptosis without the processing and release of IL-1β and IL-18.

Caspase-11 was identified almost 23 years ago in a mouse cDNA library screen for homologs of caspase-1 (25). Back then, caspase-11 was found to be a key mediator in LPS-induced septic shock model since *Casp11*^−/−^ mice are more resistant to lethal doses of LPS compared to wild-type mice (25, 26), though its mechanism of activation remained elusive. 15 years later, caspase-11 regained the center stage after Kayagaki et al. demonstrated that exposure to cholera toxin B (CTB) and certain Gram-negative bacteria, such as *Escherichia coli, Citrobacter rodentium*, and *Vibrio cholera*, activates caspase-11 to trigger pyroptosis, albeit after long exposure times (~16–24 h) (24). We subsequently showed that caspase-11 specifically differentiates Gram-negative bacteria that invade the cytosol from bacteria that remain extracellular or confined to the vacuole, with the former activating caspase-11 rapidly (27) (Figure 1). However, like the experiments before, vacuolar and extracellular Gram-negative bacteria could still activate caspase-11 after sufficient time (28–31). These observations precipitated interest in the discovery of the identity of the common non-canonical activating agonist(s) and sensor(s). Two independent studies by the Miao and Dixit groups discovered that activation of caspase-11 is induced by LPS that has gained access to the host cytosol (32, 33). Of note, it was later determined that the activation of caspase-11 by CTB in LPS-primed macrophages was triggered by the aberrant delivery of LPS by CTB to the cytosol, rather than the direct action of CTB itself (32). The fact that LPS is the cytosolic ligand that activates caspase-11 also shed light on the role of caspase-11 in septic shock, and why septic shock could occur in the absence of TLR4, the cell-surface LPS receptor (32, 33). Therefore, LPS is detected by two receptors: extracellularly or within the recycling endosomes by TLR4 and in the cytosol via caspase-11. It is also important to note that LPS also co-localizes with intracellular TLR4 in the golgi apparatus or the endoplasmic reticulum (34, 35).

Shortly afterward, the Shao lab found that caspase-11 is the direct sensor of LPS. This was in clear contrast to the caspase-1 canonical inflammasome, where sensor and executioner functions were separated into two proteins, often bridged by the adapter ASC (36). Shi et al. demonstrated that the caspase-11 inflammasome is initiated by the CARD motif of caspase-11 directly binding to the lipid A moiety of LPS. The binding of LPS to the CARD of caspase-11 not only triggers its oligomerization but also its catalytic activation (36, 37).

In humans, caspase-11 is duplicated as caspase-4 and -5, both of which have been shown to detect directly cytosolic LPS (36). The reason for this duplication remains a mystery. One distinction is that caspase-4 expression is high in unstimulated macrophages (38–40), while murine caspase-11 and human caspase-5 are expressed at low and extremely low levels, respectively, without stimulation (39–41). Upon exposure to LPS or other toll agonists, however, macrophages rapidly induce transcription of caspase-11 and caspase- 5. Indeed, caspase-11 is non-responsive in the absence of priming through interferon-β (IFN-β) or interferon-γ (IFN-γ) (24, 27–30). Thus, as one hypothesis, caspase-11 duplication may enable a new priming-independent response through caspase-4.

## Caspase-11 Cleaves GSDMD to Drive Pyroptosis

Inflammatory caspases 11 and its human homologs caspase-4 and 5 cleave the pyroptosis effector protein, GSDMD, separating its N-terminal pore-forming domain from the C-terminal repressor domain (42–44). The N-terminal domain is inserted into the plasma membrane, and then oligomerizes to form large pores in the membrane that drive swelling and membrane rupture (22, 45–47). Additionally, pore formation leads to potassium efflux, which triggers the NLRP3/ASC/caspase-1 inflammasome and subsequent processing of pro-IL-1β and pro-IL-18 to their mature forms *in vitro* (48–51) (Figure 1); however, this pathway has not yet been confirmed *in vivo*. Additionally, GSDMD pores allow inflammasomes effectors such as IL-1α, IL-1β, IL-18, and inflammatory mediators such as eicosanoids to be released into the extracellular milieu (22, 52, 53). Although membrane pores release soluble cytosolic contents, they are small enough to retain organelles and entrap bacteria to prevent their dissemination, resulting in a structure called the pore-induced intracellular trap (PIT) (54). The PIT coordinates innate immune responses via complement and scavenger receptors to drive recruitment of and efferocytosis by neutrophils that ultimately kill the invading pathogen (54). GSDMD-dependent pores will ultimately lead to membrane rupture, but before that, cells can potentially repair membrane pores to negatively regulate pyroptosis and cytokines release. Rühl et al. showed that in macrophages, this process is coordinated by the endosomal sorting complexes required for transport (ESCRT). ESCRTs are recruited to damaged membrane in a Ca^2+^ influx-dependent manner and they remove pores from the plasma membrane (55).

In a conceptual analog to macrophage PITs, the Zychlinsky lab recently found that neutrophil GSDMD can also be cleaved by the serine protease elastase to drive formation of neutrophil extracellular traps (NETs) (56). In this situation, GSDMD cleavage releases the granular proteins including elastase that may enter the nucleus, triggering NETosis, while GSDMD forms pores in the plasma membrane to release these granules and form the NET (56) (Figure 1). Interestingly, the Schroder group showed that neutrophil exposure to cytosolic LPS drives caspase-11 activation and GSDMD cleavage, leading to the extruding of antimicrobial NETs independent of elastase (57). In this case, the combined actions of GSDMD and caspase-11 drive nuclear permeabilization, chromatin relaxation, and plasma membrane rupture prior to NETs release (57). NETs can restrict bacterial replication in the cells, entrap the bacteria to prevent their dissemination and enhance direct bacteria killing through the action of neutrophil granular contents. However, an excess of NETs has been reported for several pulmonary diseases, including asthma, cystic fibrosis (CF), influenza, bacterial pneumonia, and tuberculosis (58).

Caspase-11 and GSDMD are widely expressed in hematopoietic and non-hematopoietic cells, including macrophages, neutrophils, epithelial and endothelial cells. Lung epithelial and endothelial cells express caspase-11 and were found to be sensitive to stimulation with cytosolic LPS (7, 8). Interestingly, lung epithelial cells do not express the caspase-1 inflammasomes components NLRP3 or NLRC4 (7). Therefore, the activation of these cells through the caspase-11 non-canonical inflammasome triggers only pyroptosis without the processing of pro-IL-1β or pro-IL-18 (7) (Figure 1). Activation of caspase-11 can also induces the shedding of intestinal epithelial cells after detection of intracellular *S. typhimurium* during gut infection (59), and may perform a similar function in lung epithelial cells.

## Access to the Cytosol by LPS

Access of LPS to the cytosol is a key factor during the activation of caspase-11. Cytosol -invasive pathogens such as *Burkholderia thailandensis* and its more virulent relative *Burkholderia pseudomallei* use T3SS to escape from the vacuole to the cytosol (60). This exit provides an explanation of how LPS can gain access to the cytosol. However, several Gram-negative bacteria also activate caspase-11 but are not cytosolic. Several studies showed that LPS cytosolic access can also be mediated by the activity of host factors, such as guanylate binding proteins (GBPs) (61, 62). GBPs are cytosolic GTPases that localize to the vacuole or endosome and contribute to cell-autonomous defense against cytosolic and non-cytosolic pathogens via several mechanisms including limiting intracellular bacterial survival and motility (63–66). GBPs are well conserved among vertebrates, and are found in both mice and humans (67). Genes encoding murine GBPs are clustered on chromosome 3 and are known collectively as GBPchr3 (GBP1, GBP2, GBP3, GBP5, and GBP7) or clustered on chromosome 5 (GBP4, GBP6, GBP8, GBP9, GBP10, and GBP11). In contrast, the family is reduced in humans to encode 7 GBPs genes (GBP1-GBP7) that are clustered on chromosome 1 (68–71). Intriguingly, like caspase-11, GBPs are interferon-stimulated genes, and require interferon signaling to be upregulated (62, 68). GBP-dependent effects in the activation of caspase-11 have been established in several intracellular pathogens including, *S. typhimurium, Legionella pneumophila, C. rodentium*, T3SS-negative *Pseudomonas aeruginosa, Vibrio cholerae, Chlamydia trachomatis*, and *Chlamydia muridarum* (61, 62, 72, 73). For instance, macrophages from mice that lack GBPs on Chromosome 3 exhibited less caspase-11-mediated pyroptosis in response to *S. typhimurium* and *L. pneumophila* relative to wild-type macrophages (61, 62, 74).

Whereas, the role of GBPs in general cytosolic sensing is well-established, the precise mechanism of GBPs function in LPS sensing is still the subject of ongoing research. Nonetheless, most studies suggest that GBPs are either recruited to the pathogen-containing vacuoles (PCVs), which might promote lysis of PCVs and consequent exposure of vacuolar LPS to caspase-11, or alternatively, GBPs are recruited to spontaneously ruptured bacteria-containing vacuoles or directly to cytosolic bacteria, where they act to extract or expose lipid A buried in bacterial membranes for recognition by caspase-11 (61, 62, 75, 76). In support of this latter hypothesis, human guanylate binding protein-1 (hGBP1) associates with the surface of intracellular Gram-negative bacterial pathogens after vacuolar escape (77, 78). Then, GBP1 polymerizes to coat the bacteria and initiate the recruitment of GBP2-4 to assemble a platform for caspase-4 activation (77–79). hGBP1 binds directly to LPS (77–79) and exerts a detergent-like effect of clustering LPS, which disrupts bacterial surfaces (79), thereby exposing the membrane-embedded lipid A for caspase-4 detection. In mice, GBPs can serve as a docking platform to recruit another interferon-inducible protein, IRGB10 that can co-localize to the intracellular bacteria to disrupt the bacterial cell membrane and to liberate bacterial ligands, including LPS and DNA, for innate immune recognition (80).

Virulence-associated secretion systems in Gram-negative pathogens can also drive LPS exposure in the cytosol. Gram-negative bacteria produce and release outer membrane vesicles (OMVs) during infection that are internalized by macrophages and function as vehicles that deliver LPS (and several other surface antigens) into the host cytosol (81). LPS on OMVs can trigger caspase-11 activation during *E. coli* infection. In addition, the OMVs from Gram-negative bacteria *S. typhimurium, L. pneumophila, P. aeruginosa*, and *Shigella flexneri* activate caspase-11 in a GBPchr3-dependent manner. In this case, GBPs may disrupt OMVs causing the release of LPS to cytosol where it engages caspase-11 (82).

## Caspase-11-Mediated Defense Against Gram-Negative Bacteria in the Lung

Since caspase-11 is expressed broadly in the lung tissue, caspase-11-dependent LPS-sensing is poised to play an important role in defense against Gram-negative bacteria. As noted above, pyroptosis accomplishes the dual task of restricting intracellular bacterial growth by lysing infected cells, and releasing the cytokines IL-1β and IL-18 (6), which promote host defense via pleiotropic mechanisms. IL-1α can induce neutrophil recruitment to facilitate bacterial clearance and IL-1β is best known to promote local inflammation and to recruit neutrophils to the site of infection. In turn, IL-18 is best known to induce IFN-γ secretion by natural killer cells and cytotoxic T lymphocytes (83). During systemic infection, caspase-11 is essential for host defense against cytosol-invasive bacteria, including *B. thailandensis* and *B. pseudomallei* (27). Mice deficient in caspase-11 are extremely susceptible to even mild infection with *B. thailandensis*, while wild-type mice are completely resistant to higher doses of infection (27, 39). We showed that the NLRC4 inflammasome detects *B. thailandensis*, and activates caspase-1 to trigger pyroptosis and IL-18 secretion. Curiously, we found that *in vitro, B. thailandensis* efficiently triggers both caspase-1 and−11-dependent pyroptosis in macrophages. *In vivo*, however, caspase-1-dependent pyroptosis plays only a minor role and cannot substitute for caspase-11-dependent pyroptosis. On the other hand, caspase-1-driven IL-18 was needed only to clear early infection. IL-18 promotes a robust and rapid production of IFN-γ, which primes caspase-11. This coordinated response clears even high dose infections in 1 day (39). In the absence of IL-18 signaling, *B. thailandensis* continues to replicate *in vivo* for 3 days. This prolonged bacterial burden triggers weak IFN-γ production via another undefined pathway, which is nonetheless sufficient to prime caspase-11 during medium or low dose infection and rescue the mice from lethality. In high dose infections, this pathway comes too little and too late and *Il18*^−/−^ mice succumb to infection. Intriguingly, while endogenous type I interferons were insufficient to prime caspase-11 in the absence of IFN-γ, injection of poly (I:C), a known inducer of IFN-β, successfully bypassed the IFN-γ requirement and primed caspase-11 *in vivo* (39). It is important to note that there are no examples in the literature of any pathogen–be it bacterial, fungal, viral, or parasite–in which the magnitude of protection conferred by caspase-11 inflammasomes approaches what is seen with *B. thailandensis* (84).

Caspase-11 also provides host protection in the lung in response to infection by Gram-negative bacterial pathogens, including *B. thailandensis* (7, 85), *Acinetobacter baumannii* (86), *Klebsiella pneumoniae* (87), *L. pneumophila* (88), and *L. gratiana* (88). As mentioned above, we found that *B. thailandensis* is detected by both caspase-1 and-11 in macrophages. Additionally, Wang et al. found that caspase-1 is the most important in response to *B. thailandensis in vitro*, and he showed that the absence of caspase-1 completely ablates release of IL-1β and IL-18 as well as pyroptosis of *Burkholderia*-infected macrophages. In contrast, pyroptosis, IL-1β and IL-18 of macrophages were not affected by deficiency of caspase-11. Interestingly, during pulmonary infection, mice lacking either caspase-1, 11 or GSDMD were significantly more susceptible than wild type mice to intranasal infection with *B. thailandensis* (7, 85). To account for this phenotype, Wang et al. showed that unlike macrophages, lung epithelial cells do not express caspase-1 canonical inflammasome components and are thus incompetent for caspase-1-mediated pyroptosis (7). Therefore, macrophages are protected by both caspase-1 and−11. However, lung epithelial cells solely depended on caspase-11 to restrict intracellular *B. thailandensis* replication in these cells (7).

*A. baumannii*, a pathogenic bacterium that can cause severe pulmonary infection, can activate caspase-1 through the NLRP3 inflammasome, leading to increased IL-1β release by macrophages. However, IL-1β release is partially abrogated in caspase-11-deficient macrophages, indicating that *A. baumannii* can also activate the caspase-11 non-canonical inflammasomes (86). Furthermore, caspase-11 deficiency completely ablates IL-1α secretion and reduces the cytotoxicity of *A. baumannii* in macrophages (86). Therefore, these data suggest that caspase-11 deficiency may impair the innate immune response to *A. baumannii* infection. Indeed, caspase-11-deficient mice register higher bacterial burdens in the BALF and lung tissue during *A. baumannii* infection. Additionally, caspase-11 deficiency causes mice to exhibit exacerbated pulmonary pathology due to extensive neutrophil Infiltration (86). Similarly, pulmonary infection of *Casp11*^−/−^ with *K. pneumoniae*, showed that lack of caspase-11-dependent release of IL-1α impeded neutrophil recruitment in the early stage of *K. pneumoniae* infection and was accompanied by impaired bacterial clearance as well as exaggerated pulmonary pathological changes (87). Therefore, caspase-11 functions in the lung by promoting pyroptosis to prevent the bacteria from establishing an intracellular niche in macrophages and the lung epithelial. Additionally, caspase-11 pyroptosis can drive IL-1α and IL-1β release to recruit neutrophils that eventually will efferocytose and eliminate the offending bacteria.

## Caspase-11 Hyper-Activation Drives Inflammatory Lung Diseases

Caspase-11-dependent pyroptosis and cytokines are important for combating respiratory infections. However, their excessive activation may contribute to the development of severe diseases in the lung. Acute lung injury (ALI) is a leading cause of death in bacterial sepsis (8). ALI is characterized by massive destruction of the lung endothelial barrier which results in lung edema, influx of pro-inflammatory leukocytes and sever hypoxemia (8). During endotoxemia, high concentrations of LPS may persist and aberrantly localize to the cytoplasm, triggering the hyper-activation of caspase-11 and resulting in massive pyroptosis. The hyper-activation of caspase-11 drives endotoxin shock independently of TLR4 (32, 33). During an ALI model, Cheng et al. show that the activation of caspase-11 with LPS causes severe endothelial pyroptosis that results in ALI (8). Exposure to LPS has also been shown to play a major role in asthma development (89) and recently, caspase-11 response was implicated as a critical contributor to asthma (90). Using the ovalbumin model of allergic airway inflammation, Zaslona et al. found that expression of caspase-11 is elevated in the lung of wild type mice with allergic airway inflammation. Interestingly, they found that caspase-11-deficient mice are strongly resistant to this pathology (90). However, how caspase-11 is activated in this model isn't clear. Zaslona et al. suggest that the lung is a non-sterile environment, and LPS released from naturally occurring lung bacteria might activate caspase-11, leading to airway inflammation and asthma in susceptible individuals.

In patients with cystic fibrosis (CF), *P. aeruginosa* can cause chronic airway infection that is characterized by an exaggerated pro-inflammatory cytokine response and sustained, neutrophil-dominant inflammation (91). Previous studies have demonstrated a role of NLRP3 and NLRC4 inflammasome activation in response to *P. aeruginosa* infection in CF patients (73, 92). However, *P. aeruginosa* strains isolated from chronically infected CF patients have either defective T3SS or are non-motile, enabling them to evade NLRC4-caspase-1 inflammasome detection (93–95). Balakrishan et al. showed that caspase-11 can detect these T3SS-negative *P. aeruginosa* isolates (73). However, whether this detection takes place *in vivo* to drive inflammation and disease in CF has yet to be determined.

## Out-Smarting the Caspase-11 Non-Canonical Inflammasome

Caspase-11 protects against lethal infection mediated by cytosol-invasive pathogens (27), it is particularly important for protection against ubiquitous environmental bacteria that have not evolved to escape cytosolic detection, such as the aforementioned *B. thailandensis*. However, it is not surprising that a number of organisms have evolved specific strategies to avoid the activation of this innate immune signaling pathway. These strategies include limiting LPS access to the cytosol, modifying the structural features of LPS to avoid binding to caspase-11, or suppressing caspase-11 and/or GBPs activity.

As noted above, preventing access of LPS to the cytosol using vacuolar niches or by remaining extracellular are prominent ways to avoid caspase-11 activation. For instance, *S. typhimurium* and *L. pneumophila* use T3SS and T4SS, respectively, to translocate effector proteins that work in concert to establish and maintain an intracellular vacuolar growth niche. Loss of the *S. typhimurium SifA* or *L. pneumophila SdhA* effectors causes rupture of the vacuole and release of bacteria into the cytosol (96–98). As we might expect, these two bacteria are poorly detected by caspase-11, while their isogenic mutants for these proteins are readily detected by caspase-11, with half the attenuation of the *S. typhimurium* Δ*sifA* mutant in mice explained by caspase-11 activation alone (27).

Another strategy is to modify the chemical structure of LPS to avoid detection. Caspase-11 uses its CARD domain to bind penta- or hexa-acylated lipid A moieties on LPS (32, 33, 36). As such, several tetra-acyl lipid A structures from pathogens like *Francisella novicida, Yersinia pestis*, and *S. flexenri* have been shown to evade caspase-11/4 detection (33, 99). Indeed, during pulmonary infection, bacteria often appear to exploit these structural requirements in order to evade caspase-11. For example, *F. novicida*, a Gram-negative cytosolic bacterium, is not detected by caspase-11 (33). *F. novicida* initially synthesizes a penta-acylated lipid A structure with two phosphates, but then removes the 4' phosphate and 3′ acyl chain resulting in under-acylated LPS that does not activate caspase-11 (100, 101). A similar strategy is used by *Y. pestis*, which removes two acyl chains from its lipid A upon transition from growth at 25°C to 37°C (102). Caspase-11 detects hexa-acylated lipid A from *Y. pestis* grown at 25°C, but not tetra-acylated lipid A from bacteria grown at 37°C (33). Surprisingly, Lagrange et al. found that one of the human homologs of caspase-11, caspase-4, is able to respond to tetra-acylated LPS from *F. novicida* (103), suggesting that the caspase-11 duplication in human may have evolved to accommodate a broader LPS repertoire than that of mice.

Other strategies to evade caspase-11 detection may involve directly inhibiting the caspase-11 signaling pathway, as in the case of *S. flexneri*, which uses its OspC3 effector to specifically inhibit LPS detection by caspase-4 (104). Additionally, *Shigella* delivers an array of effector factors such as the ubiquitin ligase effector IpaH9.8 to target GBPs for proteasomal degradation (64, 65). Finally, host factors also can suppress the activity of caspase-11. For instance, L-adrenaline inhibits caspase-11 inflammasome activation through the ADRA2B receptor and the intracellular cAMP metabolism pathway in mouse macrophages or human monocytes (105). It is plausible that pathogens may manipulate this host metabolic pathway to their advantage.

## Concluding Remarks

Lung tissue is a unique epithelial space constantly exposed to a variety of assaults from infectious and non-infectious stimuli. Innate immune detection of, and responses to, intracellular pathogens that invade host cells is paramount in defending against infections. The caspase-11 non-canonical inflammsome plays a key role in this response. It is widely expressed in both hematopoietic and non-hematopoietic cells of the lung, functioning as a general sensor of Gram-negative bacteria whose activation results in protective innate immune effectors including pyroptosis, NETosis, and inflammatory cytokines. Therefore, it is not surprising that bacterial pathogens have evolved several strategies to outsmart caspase-11-mediated sensing of LPS and subsequent activation. Indeed, the importance of this pathway is demonstrated by those species that have not evolved to evade caspase-11, such as *B. thailandensis*, which strongly triggers caspase-11-dependent effector mechanisms that quickly lead to sterilization of the infection. This remarkable protection highlights an important role of inflammasomes in defense against environmental pathogens. Similarly, pathogens that poorly activate the caspase-11 inflammasome can multiply to uncontrolled levels in the lung, resulting in higher levels of LPS that will eventually access the cell cytosol and hyper-activate caspase-11, leading to LPS induced lung injury and sepsis. Future studies uncovering the structural features of lipid A that activate caspase-11 and the cell-specific roles of the caspase-11 inflammasome in lung tissue could identify new therapeutic avenues to treat infection, sepsis, and immune-mediated pathologies within the lung.

## Author Contributions

CO, AV, and YA wrote the article. CO and YA contributed to designing the figure. All authors contributed to the article and approved the submitted version.

## Conflict of Interest

The authors declare that the research was conducted in the absence of any commercial or financial relationships that could be construed as a potential conflict of interest.
